# Associations Between Blood Pressure and Arterial Stiffness With Cognition: Neuroaggression or Neuroselection?

**DOI:** 10.1161/JAHA.118.010900

**Published:** 2018-10-23

**Authors:** Isabel Ferreira

**Affiliations:** ^1^ University of Wollongong Australia

**Keywords:** Editorials, arterial stiffness, blood pressure, cognition, epidemiology, Epidemiology, High Blood Pressure, Cognitive Impairment, Pediatrics

## Abstract

See Article by Lamballais et al.

Blood pressure (BP) and arterial stiffness are closely intertwined.^1^, ^2^ Indeed, although elevated BP was traditionally viewed as a determinant of arterial stiffness, in the recent decades it has become evident that higher levels of arterial stiffness may also lead to subsequent increases in BP and incident hypertension. The bidirectional nature and strength of these associations seem to vary across the life course such that, in the young, arterial stiffening may be predominantly attributable to elevated diastolic blood pressure (DBP),^1^, ^3^ whereas in middle‐aged and older individuals, arterial stiffness leads to increases in systolic blood pressure (SBP) but not DBP, and thus increases in pulse pressure.^1^ This complex chain of events is consistent with the shift in the predominant subtypes of hypertension observed throughout aging, from isolated diastolic hypertension in the young to isolated systolic hypertension in the middle‐aged and elderly.^1^, ^2^

There is increasing evidence linking arterial stiffness and BP, particularly SBP and pulse pressure to cognitive impairments and decline, among middle‐aged and older individuals.^4^, ^5^, ^6^ Indeed, the brain is a main target affected by hypertension, through structural and functional alterations that disrupt the regulation of the cerebral microcirculation, leading to hypoperfusion, white matter injury, and ischemic and hemorrhagic strokes.^7^ Evidence to support that such associations are already observed in children is largely lacking, however.

In this issue of the *Journal of the American Heart Association (JAHA),* Lamballais and colleagues examined the cross‐sectional associations of BP and arterial stiffness with cognition not only in a large population‐based sample of middle‐aged and older individuals from the Rotterdam Study (n=5187, mean age 61.8 years), but also in a large population‐based sample of children who were born a half‐century later in the same area, and included in the Generation R study (n=5853, mean age 6.2 years).^8^ While previous studies, particularly those among the young, have focused on cross‐sectional contrasts in neurocognitive test performance between normo‐ and hypertensive small groups of children,^9^, ^10^ this study investigated the associations between the whole range of measured DBP and SBP, and also pulse pressure and carotid‐femoral pulse wave velocity as markers of arterial stiffness, with cognition scores in both the young and older cohort. Cognition was operationalized differently between the 2 cohorts, to account for the age and ethnic profile differences, that is, through age‐ and sex‐specific nonverbal IQ scores in the younger cohort, and through an overall cognition g‐factor derived from principal component analysis of 5 cognitive tests’ results in the older cohort. The main findings in the older cohort were that carotid‐femoral pulse wave velocity, SBP and pulse pressure (linearly), and DBP (nonlinearly) were associated with poorer cognition, largely in line with the findings from several other (prospective) studies in middle‐aged and older populations. In the younger cohort, only DBP (linearly) was adversely associated with cognition. These findings led to the conclusion that BP may already affect cognition during early childhood, in support of a “neuroaggressive” impact of elevated BP as observed among the older cohort, albeit attributed to different pressure components. However, this association was very weak, that is, roughly a half‐point change in nonverbal IQ score per SD (7 mm Hg) change in DBP. In addition, although this estimate was derived after accounting for the children's age, sex, ethnicity, and several perinatal, maternal, and lifestyle factors, it is likely that residual confounding and/or differential biases (eg, due to self‐reported maternal factors and parent‐reported instead of objectively measured child physical activity) were not completely removed.

Given the cross‐sectional nature of the Generation R results, like that of previous studies discussed among the young,^9^, ^10^ we cannot rule out that the observed association may actually have an opposite direction. This alternative (cognition→BP), and likely stronger hypothesis has been coined *neuroselection;* that is, children with better cognitive functioning select into healthier (or avoid hazardous) behaviors and thus display healthier outcomes.^11^, ^12^ Indeed, evidence from longitudinal studies that have followed children into adulthood have shown that better cognitive function in childhood predicts healthier lifestyles (eg, reduced odds of alcohol consumption/problems^13^ and smoking,^14^ and healthier dietary and physical activity patterns^15^), as well as healthier cardiovascular risk factors such as higher cardiorespiratory fitness^11^ and lower obesity,^11^ hypertension^14^ and metabolic syndrome^16^ in midadulthood. The beneficial association between childhood cognition and cardiovascular risk factors in midadulthood have been shown to be mediated through adult behaviors and socioeconomic factors.^17^, ^18^ Altogether, and strengthened by their designs, these studies support the view that it may not be warranted to assume that the direction of the BP‐cognition associations, as often observed after middle‐ age (ie, BP→cognition) operates equally at a young age. Instead, such associations may simply reflect a chain and snowballing of events throughout the life course, with poorer lifestyles and consequent cardiovascular risk profiles (including BP) being initially triggered by lower cognitive function at a young age. Indeed, a prospective study did not show associations between BP measured at the ages of 12 and 15 years with performance on tests assessing various cognitive domains 5 years later.^19^ Still, it remains that a recent Mendelian randomization analysis using data from 6 independent genome‐wide association studies consortia and the UK Biobank sample (n=112 151) provided no evidence for causal associations from physical health factors to later life cognitive ability; however, in the other direction, higher educational attainment (as a marker for cognitive ability in youth) predicted lower body mass index, SBP, type 2 diabetes mellitus, and coronary artery disease. Despite these phenotypic cognitive→physical health associations, this study did not find evidence for causal associations between the 2 either, but the study was limited by weak instrumental variables and/or poorly measured outcomes.^20^

Understanding the causal factors at the root of disease across the life course is essential for better targeted preventive and treatment strategies. For instance, evidence from clinical trials showing that antihypertensive treatment improves cognition remains inconclusive,^7^ possibly because most antihypertensive drugs do not target arterial stiffening specifically, and thus the predominant isolated systolic hypertension subtype observed in middle‐aged and older individuals who receive these drugs.^2^ Likewise, promotion of healthier lifestyles among the young, without tailoring to their ability to comprehend and reason, may impair their successful uptake and lifelong maintenance. Hopefully, the Generation R study will enable stronger causal insights to the relationship between cognitive function and arterial stiffness/BP as it continues to follow the participants into adulthood.

## Disclosures

None.
